# mTOR as a central regulator of lifespan and aging

**DOI:** 10.12688/f1000research.17196.1

**Published:** 2019-07-02

**Authors:** David Papadopoli, Karine Boulay, Lawrence Kazak, Michael Pollak, Frédérick A. Mallette, Ivan Topisirovic, Laura Hulea

**Affiliations:** 1 Gerald Bronfman Department of Oncology, McGill University, 5100 de Maisonneuve Blvd. West, Suite 720, Montréal, QC, H4A 3T2, Canada; 2 Lady Davis Institute, SMBD JGH, 3755 Chemin de la Côte-Sainte-Catherine, Montréal, QC, H3T 1E2, Canada; 3 Maisonneuve-Rosemont Hospital Research Centre, 5415 Assumption Blvd, Montréal, QC, H1T 2M4, Canada; 4 Département de Biochimie et Médecine Moléculaire, Université de Montréal, CP 6128, Succursale Centre-Ville, Montréal, QC, H3C 3J7, Canada; 5 Department of Biochemistry, McGill University, 3655 Promenade Sir William Osler, Montréal, QC, H3G 1Y6, Canada; 6 Goodman Cancer Research Centre, 1160 Pine Avenue West, Montréal, QC, H3A 1A3, Canada; 7 Department of Experimental Medicine, McGill University, 845 Sherbrooke Street West, Montréal, QC, H3A 0G4, Canada; 8 Département de Médecine, Université de Montréal, CP 6128, Succursale Centre-Ville, Montréal, QC, H3C 3J7, Canada

**Keywords:** mTOR, aging, senescence, mitochondria, lifespan, stem cell, proteostasis, nutrient sensing

## Abstract

The mammalian/mechanistic target of rapamycin (mTOR) is a key component of cellular metabolism that integrates nutrient sensing with cellular processes that fuel cell growth and proliferation. Although the involvement of the mTOR pathway in regulating life span and aging has been studied extensively in the last decade, the underpinning mechanisms remain elusive. In this review, we highlight the emerging insights that link mTOR to various processes related to aging, such as nutrient sensing, maintenance of proteostasis, autophagy, mitochondrial dysfunction, cellular senescence, and decline in stem cell function.

## Introduction

Aging is characterized by the gradual decline in physiological functions occurring in most tissues and organisms ^1^. The acceleration of aging in specific tissues leads to a variety of disorders, including neurodegeneration, obesity, diabetes, and cardiovascular and neoplastic diseases ^2^. One of the main pharmacological interventions prolonging life span in several model organisms (that is, yeast, nematodes, fruit flies, and mice) is rapamycin ^3^. Rapamycin, a natural product isolated from *Streptomyces hygroscopicus*, was discovered on the island of Rapa Nui in 1972 ^4^. It possesses anti-fungal, immunosuppressive, and anti-cancer proprieties, which are mediated by the inhibition of its target: mechanistic/mammalian target of rapamycin (mTOR) ^5– 7^. Accordingly, mTOR has been implicated in many of the processes that are associated with aging, including cellular senescence, immune responses, cell stem regulation, autophagy, mitochondrial function, and protein homeostasis (proteostasis) ^3, 8– 10^. Finally, in some model organisms, interventions expanding life span (for example, caloric restriction, or CR) were shown to involve TOR ^3^. This article provides an overview of the role of mTOR signaling in coordinating cellular processes involved in regulation of life span, aging, and age-related pathologies and puts an emphasis on mammals.

## Regulation and functions of mTOR pathway

**Figure 1.**
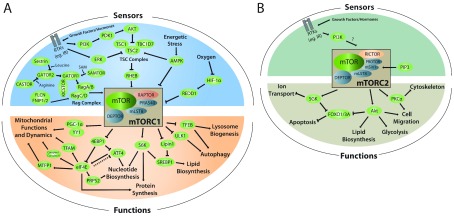
mTOR acts as a nutrient sensor coordinating cellular functions linked to proliferation, growth, and survival. mTOR operates within two functionally and structurally distinct complexes: mTORC1 and mTORC2. The two mTOR complexes share the components mLST8 and DEPTOR, while RAPTOR and PRAS40 are present exclusively in mTORC1. RICTOR, mSIN1, and Protor-1/2 are found exclusively within mTORC2. Growth factors or hormones (for example, insulin through IR) activate the PI3K/PDK1/AKT or ERK signaling pathways, which inactivate the TSC2 subunit of the TSC complex. Inactivation of the TSC complex upregulates the activity of RHEB, which in turn stimulates mTORC1. The activity of mTORC1 is also positively regulated by amino acid–mediated stimulation of the RAG complex of GTPases: Rag A/B and Rag C/D. GATOR1 inhibits RAG GTPases. SAM inhibits the activation of GATOR1 by SAMTOR. GATOR1 is also repressed by GATOR2, which in turn is regulated by Sestrin2 and CASTOR in response to leucine and arginine, respectively. The FLCN FNIP1/2 complex is also thought to stimulate the RAG activity. mTORC1 is suppressed under conditions where energy or glucose is limiting through AMPK signaling, which activates TSC2 and inhibits the mTORC1 subunit RAPTOR, and by hypoxia via the HIF-1α/REDD1 axis. mTORC1 orchestrates several anabolic processes via transcriptional or translational regulation or both. mTORC1 controls protein synthesis in part through its two main effectors: S6K and 4E-BP1. mTORC1 also stimulates mitochondrial function, through 4E-BP1 and PGC-1α/YY1, and mitochondrial dynamics (via MTFP1). The control of mTORC1-mediated nucleotide synthesis is governed by S6K, ATF4, and PRPS2 (which is translationally regulated via 4E-BP/eIF4E), while lipid biosynthesis and adipogenesis are regulated by S6K and Lipin1. mTORC1 controls autophagy by inhibiting the activity of ULK1 and TFEB; the latter also mediates mTORC1-dependent lysosome biogenesis. Conversely, the activity of mTORC2 may be regulated by growth factors through PI3K activation and generation of PIP3. PIP3 has been suggested to bind to mSin1, thereby activating mTORC2. mTORC2 activation promotes AKT signaling involved in glycolysis, lipid biosynthesis, and cell migration, while SGK signaling is involved in ion transport. Both AKT and SGK negatively regulate FOXO1/3A, which is a regulator of key metabolic pathways and apoptosis. mTORC2 also controls cytoskeleton and cell migration through PKCα. 4E-BP, eukaryotic initiation factor 4E-binding protein; AKT, protein kinase B; AMPK, AMP-activated protein kinase; ATF4, activating transcription factor 4; CASTOR, cellular arginine sensor for mTORC1; DEPTOR, DEP domain-containing mTOR-interacting protein; eIF4E, eukaryotic translation initiation factor 4E; ERK, extracellular signal-regulated kinase; FLCN, folliculin; FNIP1/2, folliculin interacting protein 1/2; FOXO1/3, forkhead box protein O1/O3; GATOR1, GTPase-activating proteins toward Rags 1; GATOR2, GTPase activating proteins toward Rags 2; HIF-1α, hypoxia-inducible factor 1 alpha; IGF1R, insulin-like growth factor 1 receptor; IR, insulin receptor; KICSTOR, KPTN-, ITFG2-, C12orf66-, and SZT2-containing regulator of mTORC1; mLST8, mammalian lethal with SEC13 protein 8; mSIN1, mammalian stress-activated protein kinase interacting protein 1; MTFP1, mitochondrial fission process 1; mTOR, mechanistic target of rapamycin kinase; mTORC1, mechanistic target of rapamycin complex 1; mTORC2, mechanistic target of rapamycin complex 2; PDK, 3-phosphoinositide-dependent protein kinase-1; PGC1α, peroxisome proliferator-activated receptor gamma coactivator 1-alpha; PI3K, phosphoinositide 3-kinase; PIP3, phosphatidylinositol (3,4,5)-triphosphate; PKC, protein kinase C alpha; Pras40, Proline-rich AKT1 substrate 1; Protor, protein observed with Rictor-1; PRPS2, phosphoribosyl pyrophosphate synthetase 2; RAG, Ras-related GTP-binding protein, subunits A/B or C/D; RAPTOR, regulatory-associated protein of mTOR; REDD1, regulated in development and DNA damage response 1; RHEB, Ras homolog, mTORC1 binding; RICTOR, rapamycin-insensitive companion of mTOR; S6K, ribosomal protein S6 kinase; SAMTOR, S-adenosylmethionine sensor for the mTORC1 pathway; SGK, serum and glucocorticoid-regulated kinase 1; SREBP1, sterol regulatory element-binding transcription factor 1; TBC1D7, TBC1 domain family member 7; TFAM, mitochondrial transcription factor a; TFEB, transcription factor EB; TSC, tuberous sclerosis complex; ULK1, Unc-51-like autophagy activating kinase; YY1, Yin Yang 1.

TOR is a serine/threonine kinase that is evolutionary conserved, and homologues are found in yeast, nematodes, flies, plants, and mammals ^11^. In higher eukaryotes, including mammals, mTOR is encoded by a single gene and its protein product is a component of two distinct complexes—mTOR complex 1 (mTORC1) and 2 (mTORC2) ^3^ —which differ functionally and structurally and in their sensitivity to rapamycin ^12– 14^ ( Figure 1). The two mTOR complexes share the components mLST8 and DEPTOR (DEP domain-containing mTOR-interacting protein), whereas RAPTOR and PRAS40 are present exclusively in mTORC1 ^3^. In turn, RICTOR, mSIN1, and Protor-1/2 are found within mTORC2 ^3^. In yeast, TOR1 and TOR2 are encoded by distinct genes; TOR2 engages in both TORC1 and TORC2 complexes, and TOR1 is exclusive for the TORC1 complex ^15^. mTORC1 responds to a plethora of extracellular stimuli and intracellular cues, such as amino acids, hormones, growth factors, energetic stress, and oxygen. These factors initiate mTOR-dependent anabolic processes, including nucleotide, lipid, and protein synthesis while inhibiting autophagy, which results in stimulation of cellular growth and proliferation ^3, 16^ ( Figure 1). Several regulators that signal via the PI3K/PDK1/AKT (phosphoinositide 3-kinase/3-phosphoinositide-dependent protein kinase 1/protein kinase B) pathway (for example, insulin and IGFs) stimulate mTORC1 by inhibiting the tuberous sclerosis complex (TSC) ( Figure 1) which is composed of TSC1 scaffold and TSC2. This complex acts as a Ras homologue enriched in brain (RHEB) GTPase-activating protein ^17^. TSC1/2 complex associates with auxiliary factor TBC1D7 (TBC1 domain family member 7) ^18^. TSC2 inactivates RHEB, leading to mTORC1 inhibition ^19, 20^. One of the major roles of mTORC1 is the regulation of protein synthesis, which is mediated via the phosphorylation of a multitude of substrates ^3, 16^, the best characterized of which are the eukaryotic translation initiation factor 4E (eIF4E)-binding proteins (4E-BPs) and ribosomal protein S6 kinases (S6Ks) ^21^. By affecting protein synthesis (that is, 4E-BPs) or by substrate phosphorylation (that is, S6Ks) or both, these factors also mediate the effects of mTOR on metabolic processes, including nucleotide synthesis, lipid metabolism (via sterol regulatory element-binding protein 1, or SREBP1), and mitochondrial function and dynamics (detailed in the “mTOR and the regulation of mitochondrial function” section) ( Figure 1). In most cell types, mTORC1 inhibition by rapamycin leads to a strong decrease in S6K phosphorylation while only marginally affecting 4E-BP phosphorylation ^22, 23^.

In contrast, mTORC2 regulates cytoskeletal organization and the activity of several members of the AGC family of kinases (for example, AKT and SGK1) and has been implicated in the degradation of newly synthesized polypeptides. It is involved in glucose and lipid metabolism through AKT-dependent and independent mechanisms ^24– 26^, controls ion transport via SGK1 ^27^, and affects the cytoskeleton and cell migration through protein kinase C alpha (PKCα) ^28^ ( Figure 1). In addition, both AKT and SGK negatively regulate FOXO1/3A (forkhead box protein O/O3A), which are transcription factors that regulate metabolism and apoptosis ^28^. In most cell lines, mTORC2 is insensitive to short-term (<24 hours) rapamycin treatment ^14, 29^, but it has been reported that mTORC2 is downregulated during prolonged rapamycin exposure in several cell lines and tissues (such as hepatocytes, adipose tissues, skeletal muscle, heart, pancreas, liver, lung, and spleen) *in vivo* ^30– 32^. Regulation of mTOR and its functions, including governing mRNA translation, is covered in detail in a number of excellent recent reviews (for example, ^3, 16, 21, 33, 34^).

## TOR as a negative regulator of life span

The relationship between TOR and longevity was first shown in non-vertebrates by using genetic manipulations. For example, deletion of S6K homologue SCH9 in *Saccharomyces cerevisiae* or depletion of TOR ( *let-363*) ^35^ or RAPTOR (mTORC1 protein member; *daf-15*) by RNA interference (RNAi) in *Caenorhabditis elegans* ^36, 37^ extends life span in both models. Similar effects were observed in *Drosophila melanogaster* either through the overexpression of TOR suppressors dTsc1 of dTsc2 or by expressing dominant-negative forms of dTOR or dS6K ^38^. Pharmacological inhibition of TOR by rapamycin in *S. cerevisiae*, *C. elegans*, *D. melanogaster*, or *Mus musculus* confirmed the evolutionary conserved and fundamental role of mTOR as a regulator of longevity ^3, 39– 47^.

### mTOR and the beneficiary effects of dietary restriction on life span

**Figure 2.**
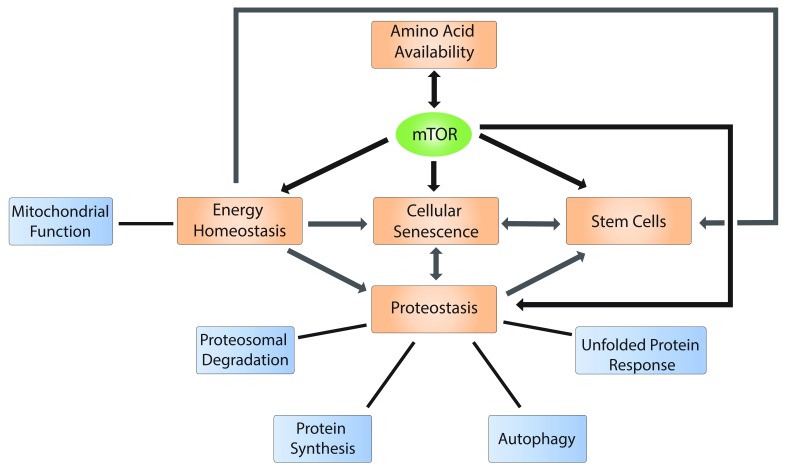
mTOR regulates several hallmarks of aging. Schematic representation of the role of the mTOR pathway in the regulation of hallmarks of aging (black arrows), such as nutrient availability (represented by amino acid availability), energy homeostasis, cellular senescence, cell stemness, and proteostasis. mTOR activity is regulated in part by amino acid levels, while mTOR in turn stimulates the synthesis of non-essential amino acids (see the “mTOR and the beneficiary effects of dietary restriction on life span” section). The depicted hallmarks of aging are also interconnected (grey arrows), suggesting that aging is a coordinated process in which mTOR plays a significant role. mTOR, mechanistic target of rapamycin kinase.

The central role for the mTOR pathway in regulating life span has been attributed in part to its function as a nutrient sensor ( Figure 2). Nutrient-sensing pathways, including the insulin/insulin-like growth factor 1 (IGF-I) signaling (IIS) network, are thought to act as major determinants of longevity. The importance of IIS, like mTOR pathway inhibition, in regulating life span was firmly established in numerous species ( *C. elegans*, *D. melanogaster*, or *M. musculus*) ^48– 52^. IIS activation via binding of insulin or IGF-I to the insulin receptor (IR) or IGF-1 receptor (IGF1R) (or both) leads to activation of PI3K/AKT. Signaling through additional growth factor receptors (for example, epidermal growth factor receptor family, or ERBB) activates the RAS/RAF/MEK/ERK pathways. As AKT, ERK, and its downstream effector RSK1 all phosphorylate and inactivate TSC2 ^53– 56^, PI3K and RAS/RAF/MEK/ERK pathways largely converge on mTORC1 ( Figure 1) ^3^.

Suppression of the IIS/mTOR axis is thought to be one of the underpinning mechanisms of the beneficiary effects of dietary restriction (DR), which not only prolongs life span but also delays the onset of age-related pathologies (commonly referred to as health span) across a variety of organisms (yeasts, nematodes, fruit flies, rats, mice, and primates) ^57– 59^. DR incorporates both the classic concept of CR (where the total caloric intake is reduced, usually by 20 to 40%) and restriction of specific nutrients or regimens of restriction for intermittent time periods ^60^. Consistent with the central role of mTOR in nutrient sensing, CR did not further prolong life span under conditions in which TORC1 was inhibited by TOR1 deletion in *S. cerevisiae*, TOR RNAi in *C. elegans*, or overexpression of dTsc2 in *D. melanogaster* ^38, 61, 62^. Conversely, several reports suggested that rapamycin may potentiate the effect of CR in increasing life span in *D. melanogaster*, indicating potential complexity in the role of mTOR inhibition in mediating the beneficiary effects of CR ^40^. Comparison of the effects of rapamycin and CR in liver and white adipose tissues in mice ^63, 64^, or experiments carried out in *S. cerevisiae* ^65^, revealed that rapamycin and CR induce discrete changes in transcriptome and metabolome, suggesting that CR may extend life span through mTOR-independent mechanisms. Notably, rapamycin incompletely inhibits some of mTORC1 substrates, including 4E-BPs ^22, 23, 66^. This is particularly significant when comparing the impact of mTOR inhibition and CR on metabolome, as 4E-BPs mediate mTORC1-dependent translational regulation of several metabolic processes (for example, cellular energetics, mitochondrial dynamics, and non-essential amino acid synthesis) ^67– 69^ ( Figure 1). Translatome studies thus are warranted to grasp the full overlap between the effects of CR and mTOR on life span and aging.

As CR is not readily translatable to the clinic, alternative DR strategies based on macronutrient restrictions have been tested. In these approaches, specific macronutrient intake is limited without a reduction in calories ^60^. DRs were shown to improve healthy aging in humans; one of the most effective interventions appears to be the reduction of protein and amino acid intake (protein restriction, or PR) ^70– 72^. Indeed, PR extends both life span and health span in mice but was linked to reductions in cancer, diabetes, and overall mortality in humans ^70^. In some cases, the reduction of a single amino acid, such as methionine or tryptophan, was able to cause these effects ^73– 82^. In addition to methionine deprivation, restriction of leucine and other branched-chain amino acids (BCAAs) (isoleucine and valine) improved metabolic health (such as better glucose tolerance and reduced fat mass gain) in both normal and obese mice ^83– 85^. Conversely, long-term exposure of mice to an isocaloric yet high BCAA-containing diet led to hyperphagia and obesity and reduced life span ^86^. Interestingly, the mechanism was not related to increased total BCAA intake or high mTORC1 but to an amino acid imbalance (shift in the relative quantity of dietary BCAAs versus other amino acids such as tryptophan and threonine) ^86^. Metabolites derived from amino acids might also influence life span. Recent evidence indicates that homocysteine, a component of the methionine cycle, can activate mTORC1 ^87^. Moreover, given that amino acid availability is a major regulator of mTORC1 ^3^ (detailed in the “mTORC1 as a sensor of amino acids” section; Figure 2) and that mTOR inhibition induces increased life span, it is plausible that the beneficial effects of PR on life span and health span are mediated via mTOR. Although this has yet to be determined in the context of aging, a study using a breast cancer xenograft model showed that PR inhibits tumor growth, while reducing mTORC1 (but not mTORC2) activity in both tumor and normal tissues ^88^.

### mTORC1 as a sensor of amino acids

Nutrient sensing is often dysregulated in aging cells ^1^ ( Figure 2). Amino acids act as pivotal mTORC1 regulators ^3^ and indicators of nutrient availability. Indeed, mTORC1 is a central sensor of amino acid availability, which regulates cellular and organismal energetics ^89– 91^. In mammals, mTORC1 activation by amino acids is mediated by Ras-related GTP binding (RAG) GTPases ^92, 93^ which form A/B and C/D heterodimers that associate with lysosomal membranes via the Ragulator complex, also known as LAMTOR (late endosomal/lysosomal adaptor, MAPK and MTOR activator) ^94^. Active RAG heterodimers (in which RAG A/B and RAG C/D are bound to GTP and GDP, respectively) recruit mTORC1 to the lysosomal surface, where mTORC1 becomes activated by RHEB ^95^. As observed for mTORC1, RHEB localization is also modulated by amino acid signaling, which promotes amino acid–dependent interaction of RHEB with microspherule protein 1 (MCRS1), leading to its maintenance at the lysosome ^96^. RAGs are controlled mainly by the GATOR1/2 interplay. GATOR1, a complex containing DEP domain containing 5 (DEPDC5), nitrogen permease regulator 2-like (NPRL2), and nitrogen permease regulator 3-like (NPRL3) proteins, acts as a GTPase-activating protein (GAP) toward RAGs A/B, thereby negatively regulating mTORC1 ^97^. GATOR1 recruitment to the lysosomal surface in the context of amino acid or glucose deprivation is mediated by KICSTOR, a complex composed of kaptin, actin binding protein (KPTN), integrin alpha FG-GAP repeat containing 2 (ITFG2), chromosome 12 open reading frame 66 (C12orf66), and SZT2 ^98^. In turn, GATOR2, which is composed of MIOS (meiosis regulator for oocyte development), WD repeat domain 24 (WDR24), WDR59, SEH1-like nucleoporin (SEH1L), and SEC13, suppresses GATOR1, thus activating mTORC1 ^97^. In addition, the folliculin (FLCN)-folliculin-interacting protein (FNIP) complex was shown to display GAP activity toward RAG C/D, therefore contributing to RAG heterodimer complete activation in response to amino acids ^99, 100^. However, it is worth mentioning that FLCN loss does not always result in mTORC1 inhibition, suggesting a context-dependent function for FLCN in TORC1 signaling or the presence of compensatory mechanisms leading to mTORC1 activation in the absence of FLCN (discussed in 100, 101).

It appears that the mechanisms of activation of mTORC1 are amino acid–specific. To this end, lysosomal amino acid transporter SLC38A9 is necessary for the arginine-dependent activation of mTORC1 via its interaction with the RAG-LAMTOR-v-ATPase (vacuolar-type H + ATPase) complex ^102– 105^. In parallel, mTORC1 downregulation in response to arginine deprivation was shown to require cellular arginine sensor for mTORC1 (CASTOR1) ^106^, which interacts with GATOR2 to inhibit its function ^107^. Conversely, arginine binding by CASTOR1 triggers its dissociation from GATOR2 and consequently activates mTORC1 ^106, 107^. Through a similar mechanism, Sestrin2 functions as a direct leucine sensor ^108^ by binding to and repressing GATOR2 in the absence of leucine ^109, 110^. Upon binding to leucine, Sestrin2 dissociates from GATOR2, which leads to mTORC1 activation ^108– 112^. It has been reported that, in addition to Sestrin2, leucyl-tRNA synthetase (LRS) participates in leucine-dependent modulation of mTORC1 signaling ^113^. It has been proposed that LRS positively regulates the RAG GTPase cycle by acting as a GAP toward RAG D ^113– 115^, although this model has been challenged by other reports ^100, 116^. A recent study evidenced LRS-mediated leucylation on lysine residues of RAG A/B, resulting in mTORC1 activation ^117^. Methionine availability is indirectly sensed by the SAMTOR protein, which can bind to S-adenosylmethionine (SAM), a metabolite derived from methionine. SAM disrupts the SAMTOR–GATOR1 association and thus relieves the inhibitory effect of this complex on mTORC1 signaling ^118^. Finally, glutamine-dependent activation of mTORC1 is dependent on v-ATPase and also requires the adenosine diphosphate ribosylation factor 1 (ARF1) GTPase ^119^. Although a number of studies show that alterations of these newly discovered mTOR regulators (for example, Sestrin2) may play a role in aging ^120– 122^, their precise effects on life span and aging-related pathologies remain to be established.

### mTOR as a regulator of food intake and global energy homeostasis

The mechanisms by which CR and DR expand life span are complex and comprise organismal level regulation, including sensory food perception and modulation of food intake behaviour. For example, recent work in *C. elegans* showed that activation of a food deprivation signal through chemical manipulation mimics a state of DR, leading to prolonged life span ^123^. Food intake behaviour is in part controlled by neurons in the mediobasal hypothalamus (MBH), a key region of the brain implicated in regulation of energy balance ^124^. In MBH neurons, mTORC1 activity is induced by food intake and repressed by fasting ^125^, thereby suggesting that mTORC1 plays a pivotal role in the hypothalamic regulation of energy balance ^126, 127^. Several studies, however, failed to confirm the role of mTOR in the regulation of energy balance or feeding behaviour ^91, 128^ though confirming mTORC1 function in regulating glucose metabolism ^128^. Importantly, overexpression of DEPTOR (either systemic or restricted to MBH neurons) prevents obesity induced by a high-fat diet and improves glucose metabolism ^129^. DEPTOR is a component of both mTORC1 and mTORC2 and is able to suppress the activity of both complexes ^130^. In addition, mTORC1 in agouti-related protein (AGRP) neurons (part of the MBH) appears to facilitate integration of food-related cues and adaptive energy expenditure ^131^. This supports a role for mTOR in regulating systemic energy homeostasis ^91^, which may have an impact on metabolic disorders and aging. Moreover, in addition to its activity in MBH neurons, alterations of the mTORC1 pathway in the liver and adipose tissue were shown to impact whole-body metabolism via peroxisome proliferator-activated receptor gamma coactivator 1 alpha (PGC-1α, a transcriptional coactivator controlling mitochondrial biogenesis ^132^) and fibroblast growth factor 21 (FGF21, a secreted protein that is a key mediator of fatty acid oxidation and lipid metabolism ^133^), respectively ^134^. Finally, RAPTOR knockout (KO) in the adipose tissue of mice resulted in lean phenotype which was accompanied by elevated mitochondrial respiration ^135^. In conclusion, although these findings position mTOR as a central metabolic regulator at the cellular or whole-organism level or both, the precise relationship between these mTOR functions and life span, health span, and aging is incompletely understood.

## The role of mTOR in age-associated processes (hallmarks of aging)

In an attempt to define common denominators of aging, the following nine “hallmarks” have been proposed: genomic instability, telomere attrition, epigenetic alterations, loss of proteostasis, deregulated nutrient-sensing, mitochondrial dysfunction, cellular senescence, stem cell exhaustion, and altered intercellular communication ^1^. Not surprisingly, in keeping with the observations implicating mTOR in increasing life span, a significant proportion of these “hallmarks” are known to be affected by mTOR. In this section, we describe recent advances linking mTOR to these key age-associated processes and explore how aberrant mTOR signaling may orchestrate their coordinated dysregulation.

### Loss of proteostasis

Alterations in protein production, degradation, folding, and trafficking—proteostasis—are linked to aging ^136^. For instance, the chronic expression and accumulation of misfolded or aggregated proteins lead to the development of various age-associated diseases (for example, Alzheimer’s disease [AD] and Parkinson’s disease and cataracts) ^137^. Proteostasis is maintained via orchestration of protein synthesis, protein clearance (via proteasomal degradation or autophagy), and quality control mechanisms (for example, unfolded protein response, or UPR), all of which are thought to be influenced by mTOR. In model organisms, including *S. cerevisiae*, *C. elegans*, and *D. melanogaster*, deletion of different components of the translational machinery and subsequent decrease in protein biosynthesis significantly increase life span ^138^. mTORC1 stimulates protein synthesis through several mechanisms that have been highly characterized and that involve two main mTORC1 effectors: S6K and 4E-BPs ^21^. mTORC1 also suppresses autophagy, in part by inhibiting Unc-51-like autophagy-activating kinase (ULK1) ^139^. It is now emerging that protein synthesis and autophagy, coupled with nutrient sensing, are coordinated by mTORC1 to maintain energy and protein homeostasis ^139^ ( Figure 2). In addition to autophagy, mTOR may suppress protein degradation by controlling proteasomal activity ( Figure 2). For example, mTORC1, but not mTORC2, inhibition increases protein degradation by the proteasome by enhancing ubiquitination through a mechanism that remains to be elucidated ^140^. Moreover, in yeast and mammalian cells, TOR/mTOR inhibition upregulates the levels of the regulatory particle assembly chaperones (RACs) through the activity of MAPKs Mpk1 (in yeast) or ERK5 (in mammalian cells), and increases proteasome abundance ^141^. These findings contradict previous reports that mTORC1 inhibition reduces proteolysis by suppressing expression of proteasomal subunits in a nuclear respiratory factor 1 (NRF-1)-dependent manner ^142^.

To maintain proteostasis under stress, cells engage quality control mechanisms, such as the UPR, which plays a major role in aging in both cell-autonomous and non-autonomous manners ^143^. The activation of the UPR declines with age, as was shown in multiple model organisms ( *C. elegans*, *M. musculus*, or *Rattus norvegicus*) ^143^. Conversely, UPR activation can extend life span ^143^. The UPR is initiated through the activation of three arms: inositol-requiring enzyme 1 (IRE1), PKR-like ER kinase (PERK), and activating transcription factor 6 (ATF6) ^144^. ATF6 and IRE1 increase abundance and splicing of the mRNA encoding X-box binding protein 1 (XBP1), respectively ^145– 147^. XBP1 is a transcription factor that controls the expression of genes implicated in protein folding, quality control, and lipid biosynthesis ^143^. IRE1 also activates stress signaling, including JNK and nuclear factor kappa-light-chain-enhancer of activated B cells (NF-κB) ^148^. Rapamycin suppresses IRE1-dependent JNK induction, XBP1 splicing, and activation of PERK ^149, 150^, which suggests significant cross-talk between mTOR and UPR.

The integrated stress response (IRS) is the arm of the UPR which is under governance of PERK ^151^. It is centered on the α-subunit of eIF2, which is phosphorylated by four eIF2α kinases—PERK, double-stranded RNA-dependent protein kinase (PKR), heme-regulated eIF2α kinase (HRI), and general control non-derepressible 2 (GCN2)—in response to ER stress, viral infection, heme insufficiency, and amino acid depletion, respectively ^7^. eIF2 delivers initiator tRNA (tRNA _i_^Met^) as part of the eIF2:GTP:tRNA _i_^Met^ ternary complex (TC), whereby upon tRNA _i_^Met^ delivery GTP is hydrolyzed to GDP ^33^. A multi-subunit guanine nucleotide exchange factor (GEF) eIF2B exchanges eIF2-bound GDP to GTP, which allows recycling of TC to facilitate the next initiation round ^152, 153^. Phosphorylation of eIF2α suppresses the GEF activity of eIF2B, which limits TC recycling ^154^. This results in downregulation of global protein synthesis with concomitant translational activation of mRNAs which contain inhibitory upstream open reading frames (uORFs) in their 5′ untranslated regions (5′UTRs) ^155^. Transcripts that contain uORFs are enriched in those encoding stress-responsive transcription factors, including activating transcription factor 4 (ATF4) and CCAAT enhancer-binding protein homologous protein (CHOP) ^156– 158^. As is the case for UPR, several cross-talk mechanisms between ISR and mTORC1 have been observed. For example, during chronic ER stress, which leads to significant reprogramming of translational machinery that is distinct from the acute ISR ^159^, eIF2α phosphorylation is paralleled with the ATF4-dependent transcriptional induction of amino acid transporter expression ^160^ which increases intracellular amino acid levels, thus elevating mTORC1 activity ^161^. However, long-term stress may also decrease protein synthesis via the suppression of mTORC1-mediated translational control, through an AKT-dependent, eIF2α phosphorylation-independent mechanism ^161^, highlighting the fine-tuned regulation of translational activity in conditions of stress. In turn, upon nutrient, growth factor, or hormone stimulation (or a combination of these), mTORC1 in concert with casein kinase II (CK2) phosphorylates the eIF2β subunit of eIF2. eIF2β recruits the non-catalytic region of tyrosine kinase adaptor protein 1 (NCK1) that serves as an adaptor for the protein phosphatase 1 (PP1), thereby leading to eIF2α dephosphorylation ^162^. mTORC1 inhibition is also paralleled by GCN2 activation and increased eIF2α phosphorylation through a mechanism dependent on protein phosphatase 6 (PP6C) ^163^. In addition, in cells characterized by the loss of its negative regulator TSC, mTORC1 downregulates eIF2α phosphorylation by attenuating PERK activity, which is accompanied by increased reactive oxygen species (ROS) production ^164^. Finally, mTORC1 can influence ISR via regulation of ATF4 protein levels by stabilizing and promoting translation of ATF4 mRNA, which is mediated by 4E-BP1 ^165^. Given the central role of UPR and in particular ISR in aging, these data suggest that the aging-related effects of mTOR may be influenced at least in part by its cross-talk with the UPR/ISR machinery.

### mTOR and the regulation of mitochondrial function

Aberrant mitochondrial function is a major characteristic of aging cells ^1^. For example, genetic models of mitochondrial dysfunction (such as mutation of mitochondrial DNA polymerase in mice) correlate with reduced life span ^166^. However, many interventions that extend life span (CR and rapamycin) are associated with reduced energy intake and decreased mitochondrial functions ^167^. These apparently contradictory findings revealed complex and antagonistic functions of mitochondria in aging and have been at least partially reconciled by a biphasic modeling of mitochondrial dysregulation ^167^. This model proposes that alterations of mitochondrial functions are not linear with the aging of the organism but rather that they increase and peak in middle age, followed by a decline in older age ^167^.

Mitochondrial dysfunction in aging cells is characterized by several factors, including elevated ROS production and mitochondrial DNA mutations; decreased electron transport function, membrane potential, and ATP production; altered mitochondrial dynamics; or dysregulated mitophagy ^1, 168^. Functionally, altered mitochondrial activity participates in inducing cellular senescence ^169^, chronic inflammation, and a decline in stem cell activity associated with aging ^1^ ( Figure 2).

mTORC1 regulates mitochondrial biogenesis, functions, and dynamics through translational and transcriptional mechanisms ( Figure 1). mTORC1 stimulates translation of nuclear-encoded mitochondria-related mRNAs—for example, components of complex I and V, mitochondrial ribosomal proteins, and mitochondrial transcription factor a (TFAM) ^68, 170^—and fission process 1 (MTFP1) mRNA ^69^. mTORC1 activity therefore stimulates mitochondrial respiration and ATP production to meet the high energetic requirements of cancer cells ^68^. Translational suppression of MTFP1 levels leads to mitochondrial hyperfusion and protection from apoptosis ^69^. In addition to these translational mechanisms which are thought to be engaged during acute activation of mTORC1 (12 hours or less), it has been shown that, during prolonged stimulation, mTORC1 regulates transcription of nuclear-encoded mitochondrial genes by engaging PGC-1α and transcription factor Yin-Yang 1 (YY1) ^171^. mTORC2 was also shown to play a role in mitochondrial functions in both mouse embryonic fibroblasts and cancer cells, and its suppression was paralleled by increases in mitochondrial membrane potential, ATP production, and calcium uptake ^172^.

Mitophagy is the selective degradation of mitochondria by autophagy that serves as a quality control mechanism to ensure recycling and removal of damaged mitochondria ^173^. Several studies have shown that mitophagy can extend life span ^174, 175^. Interestingly, it was recently revealed that, in addition to having an established role in inhibiting autophagy ^139^, mTORC1 is involved in the regulation of mitophagy ^176^. In TSC2-null cells, in which mTORC1 is hyperactive, the levels of mitophagy induced by a mitochondrial uncoupling agent were reduced ^176^. This was accompanied by an mTORC1-dependent decrease in PTEN-induced kinase 1 (PINK1) levels and a decrease in PARK2 translocation to the outer mitochondrial membrane, which are thought to be essential for the degradation of uncoupled mitochondria by mitophagy ^176^.

Collectively, these findings implicate mTOR in many aspects of mitochondrial functions, biogenesis, degradation, and dynamics. However, more work is still needed to establish the extent of mTOR involvement in mitochondrial dysfunction in aging cells and how this is linked to the aging phenotype and orchestrated with other aging hallmarks (for example, cellular senescence).

### The role of mTOR in cellular senescence

Cellular senescence is an evolutionary conserved phenomenon characterized by a permanent and stable cell cycle exit ^177^. Senescent cells are characterized by an increase in cell size and mitochondrial mass, mitochondrial dysfunction, and the development of a multi-component senescence-associated secretory phenotype (SASP; comprising growth factors, cytokines, metalloproteases, etc) ^1, 178– 181^. Cellular senescence can be induced in response to a variety of stresses and signals (oncogenes, DNA damage, telomeric dysfunction, and oxidative stress) ^182– 186^ and, depending on the context, can be either beneficial (for example, in wound healing) ^187^ or deleterious (for example, during aging) ^188– 190^. The deleterious effects exhibited in aging are strongly linked to the SASP, whose components include a large number of cytokines, chemokines, growth factors, and proteases promoting inflammation, angiogenesis ^191^, tissue degeneration, and tumor growth ^192^. This explains why the accumulation of only a small percentage (2 to 3%) of senescent cells in tissues can have a significant negative impact ^192^. Owing in large part to SASP and the need to produce and secrete large quantities of factors, senescent cells are highly metabolically active and show high levels of protein synthesis ^193^. mTOR plays an important role in promoting the secretory phenotype of senescent cells ^194^ and its inhibition was shown to prevent stem cell senescence ^8, 9^. For example, rapamycin was shown to reduce interleukin 1 receptor (IL-1R)-dependent SASP by inhibiting the translation of IL-1α mRNA, which reduces transcription of inflammatory genes regulated by the pro-inflammatory transcription factor NF-κB ^195^. Moreover, mTORC1 is thought to interact with MAPK to increase translation of the MK2 kinase (MAPKAPK2), which prevents the degradation of numerous SASP factor transcripts by ZFP36 ring finger protein-like 1 (ZFP36L1) ^196^. Inhibition of mTOR by rapamycin was observed to impede increases in mitochondrial DNA, biomass, and ROS associated with genotoxic stress-induced senescence ^197^. Interestingly, mTORC1 activation in senescent cells may be the result of defects in amino acid and growth factor sensing ^198^. Namely, it was observed that the induction of senescence in human fibroblasts by stress, replicative exhaustion, or oncogene activation renders mTORC1 constitutively active and insensitive to serum and amino acid starvation ^198^.

Importantly, several other age-associated hallmarks such as mitochondria dysfunction and dysregulation of proteostasis are directly linked to cellular senescence, as both a cause and a consequence ^177^. Given the central role of mTOR in regulating the main aspects of these hallmarks of aging, it is plausible that aberrant mTOR signaling underlies their concerted dysregulation ( Figure 2).

### The role of mTOR in stem cell maintenance and stem cell function decline in aging

Adult tissues contain populations of somatic stem cells (also referred to as adult stem cells), which allow their regeneration under normal physiological conditions (for example, due to cellular turnover) and during response to injury ^199^. Adult stem cells are able to self-renew and differentiate into multiple cell types and they reside in specialized niches within the tissues ^199^. However, they can accumulate mutations or undergo epigenetic changes (or both) which may compromise their functions or lose the ability to divide (a phenomenon referred to as stem cell exhaustion) ^199^. To avoid this, adult stem cells are maintained in a quiescent state until activated ^199^. Quiescent stem cells are characterized by a reduction in metabolic, transcriptional, and translational activity, which correlates with a suppression of mTORC1 ^199, 200^. Depending on the tissue type, stem cells accumulate with age (for example, hematopoietic stem cells [HSCs] or intestinal stem cells [ISCs]) or are reduced in number (for example, neural stem cells [NSCs] or germline stem cells) ^201– 203^. Importantly, stem cells in aging tissues show impaired stem cell functions because of cell-intrinsic factors (accumulation of DNA damage, epigenetic alterations, and ROS; mitochondrial dysfunction; and loss of proteostasis) and extrinsic factors (disruption of the niche and chronic inflammation) ^204^, which ultimately lead to impaired tissue regeneration.

mTOR has been implicated in the self-renewal, proliferation, and differentiation of both embryonic and adult stem cells (reviewed in 205). Although the role of mTORC1 in these processes is better established ^205^, the importance of mTORC2 is also observed in promoting osteoblast and inhibiting adipocyte differentiation, reducing neural progenitor cell proliferation, and promoting HSC formation from endothelial cells ^205^. Collectively, these studies suggest that increased mTOR activity can be both beneficial and detrimental to stem cells, depending on the cellular context. For example, it was shown that CR in mice causes Paneth cells in the ISC niche to secrete cyclic ADP ribose, which in turn activates SIRT1 in ISCs ^206^. Contrary to what is expected under CR, this increases mTORC1 activity toward S6K1 and protein synthesis, thus resulting in higher ISC numbers ^206^. mTOR was also shown to be beneficial in NSCs in the hippocampus. Indeed, the mTOR pathway is compromised in aged NSCs, while its activation can revitalize the NSCs by increasing proliferation and promoting neurogenesis ^207^. By contrast, in *Ercc1* ^−/Δ^ mice, in which DNA damage repair is compromised (thus leading to premature aging), mTOR signaling is activated in muscle-derived stem/progenitor cells (MDSPCs) ^208^. Rapamycin treatment stimulates autophagy and improves the myogenic differentiation capacity of the *Ercc1* ^−/Δ^ MDSPCs, suggesting that in this context hyperactive mTOR contributes to stem cell dysfunction ^208^. Finally, in S6K1 ^−/−^ mice that show extended life span, age-associated decrease in HSC function was improved, as compared with wild-type mice ^209^, suggesting that increased mTORC1 signaling may decrease the HSC function during aging.

Although there have been great advances in dissecting how the mTOR pathway regulates various aspects of adult stem cell function, it is becoming apparent that this role of mTOR is dependent on the cellular and tissue context. Moreover, the role of mTOR in coordinating stemness with other hallmarks of aging is still largely unknown. For instance, senescence-associated reprogramming was shown to promote cancer stemness ^190^, whilst impairment in cell stemness is facilitated by mitochondrial and proteostatic dysfunctions and is accompanied by an altered metabolic state ^199^.

## The role of mTOR in aging tissues

Although the modulation by mTOR of the hallmarks of aging described above—see the “TOR as a negative regulator of life span” and “The role of mTOR in age-associated processes (hallmarks of aging)” sections—could be generalized to many cellular and tissue types, several studies to date have investigated the role of the mTOR pathway in the aging of specific tissues. Some examples are provided below.

### Heart tissue

mTORC1 is necessary for normal cardiovascular development, maintenance, and function at both the embryonic and postnatal states ^210^. This was demonstrated in various mouse models carrying constitutive or inducible cardiac tissue-specific deletions of mTOR, RAPTOR, RICTOR, or RHEB ^211– 215^. Nonetheless, mTORC1 suppression seems to be beneficial in aging cardiac tissue ^210^, as rapamycin treatment in 24-month-old mice showed improved cardiovascular function and a reversal or attenuation of age-related heart pathologies (heart inflammation and cardiac fibrosis) ^216^. RNA sequencing analysis suggested that these benefits were linked to changes in inflammatory, metabolic, and anti-hypertrophic profiles ^216^. Moreover, both CR and rapamycin treatments significantly reversed age-associated proteomics changes observed in old hearts ^217^, which were characterized by reduced abundance of proteins involved in mitochondrial functions, electron transport chain, the citric acid cycle, and fatty acid metabolism ^217^.

### Central nervous system

Similar to cardiac tissue, mTOR is required for normal neurological development and function (synaptic plasticity, neuroendocrine regulation, and neuronal recovery) and for adequate cognitive function ^218– 220^ but is dysregulated in older tissue ^220, 221^. Although mTOR activity had been found to be downregulated in the aged versus mid-age naked mole rats (as evidenced by the p-mTOR/mTOR ratio) ^222^, the mTOR pathway was shown to be hyperactivated in AD in both mouse models and humans ^221^. AD is characterized by a reduction in autophagy (and loss of proteostasis in general), impaired glucose metabolism, and decreased mitochondrial functions, all of which are governed by mTOR (as discussed in the “Loss of proteostasis” and “mTOR and the regulation of mitochondrial function” sections and 10). When administered in the early stages of the disease, rapamycin or rapalogs (rapamycin analogs) were shown to prevent cognitive decline in mouse models of AD ^223– 226^, which correlated with a decrease in aggregated beta-amyloid plaques, tau tangles, and microglia activation, all main characteristics of AD ^224– 226^. In addition, a recent study tested the use of a novel rapamycin intranasal administration protocol (InRapa), aimed at maximizing brain delivery while reducing systemic side effects ^227^. In a mouse model of Down syndrome, InRapa administration ameliorated Alzheimer-like cognitive decline, in part by rescuing autophagy and attenuating dysregulated insulin signaling ^227^. Intriguingly, in human trials of immunosuppression after heart transplantation, patients taking the rapalog everolimus showed improved memory and concentration compared with the control group ^228^. Collectively, these new findings suggesting the use of mTORC1 inhibitors for improving cognitive function and neurogenerative disorders, combined with improved strategies for drug delivery that reduce side effects, represent promising therapeutic perspectives for the future.

### Adipose tissue

There are two major adipose tissues: white adipose tissue (WAT), which stores energy in the form of triglyceride droplets and mediates energy status signaling to the hypothalamus ^229^, and brown adipose tissue (BAT), which can dissipate energy in response to cold exposure and caloric excess through coupled and uncoupled respiration and heat production ^230– 232^. Therefore, BAT confers protection from obesity and other metabolic diseases ^231, 233^. BAT also shows increased mitochondrial density and activity compared with WAT ^234^. A third population of adipocytes was recently identified, called beige adipocytes ^235^, which has characteristics of BAT and can be found in clusters within WAT. With age, there is a decline in both BAT and the “browning” of WAT (the appearance of thermogenic adipocytes within WAT depots) ^236^. The importance of BAT in aging was highlighted by recent findings showing that regulator for G protein signaling 14 (RGS14) KO mice have an extended life span that is associated with an increase in BAT, protection against cold exposure, and improved metabolism ^237^. Importantly, transplantation of BAT from RGS14 mice exerts the same protective effect in wild-type recipient mice ^237^. The TSC1/mTORC1 axis was shown to control the BAT-to-WAT phenotypic switch in adipocytes, whereas rapamycin treatment reverses the adipocyte phenotypic switch ^238^. Moreover, adipose-specific depletion of RAPTOR, which leads to a lean phenotype with enhanced mitochondrial respiration ^135^, also promotes beige adipogenesis through prostaglandins (PGs) synthesized by cyclooxygenase-2 (COX-2) ^239^. Mechanistically, COX-2 is negatively regulated by mTOR via the phosphorylation of CREB-regulated transcription coactivator 2 (CRTC2) ^239^. DEPTOR (inhibitor of mTOR and part of mTORC1) is also a regulator of adipogenesis inasmuch as its reduction of mTORC1-mediated feedback inhibition of insulin signaling activates the proadipogenic Akt/PKB-PPAR-γ axis ^240^. Indeed, DEPTOR has elevated expression in WAT of obese mice whereas in humans DEPTOR expression in WAT correlates with the degree of obesity ^240^.

In light of the protective roles of BAT and adipocyte browning against aging phenotypes, it can be speculated that the positive effects of mTORC1 inhibition on BAT and the stimulation of beige adipogenesis contribute to the anti-aging effects of rapamycin. This is plausibly also linked to the organismal energy status signaling capacity of BAT and the central role that mTOR plays in nutrient and energy sensing (detailed in the “mTORC1 as a sensor of amino acids” and “mTOR as a regulator of food intake and global energy homeostasis” sections). More work is necessary to understand how these complex interplays of networks and tissues are dysregulated in aging organisms.

### Skeletal muscle

Aging skeletal muscle is characterized by muscle fiber loss leading to atrophy (sarcopenia). Although mTORC1 signaling is needed for increased muscle mass in response to exercise or during tissue repair ^241^, very recent studies showed that mTORC1 signaling is activated in a subset of skeletal muscle fibers in aging mouse and human, which paradoxically was associated with fiber damage ^242^. In addition, hyperactivation of mTORC1 (such as in TSC1 KO mice) led to abnormal mitochondria, oxidative stress, and damage and loss of fibers. The mechanisms involved mTORC1 regulation of STAT3 phosphorylation, associated with an increase in the expression of growth differentiation factors (GDF 3, 5, and 15) and of genes involved in oxidative stress and mitochondrial catabolism ^242^. These processes were reversed by mTOR inhibition ^242^. Moreover, as discussed in the “role of mTOR in stem cell maintenance and stem cell function decline in aging” section, in a model of premature aging, mTOR activity is increased in MDSPCs and causes stem cell dysfunction ^208^. This indicates that maintaining a low basal state of the mTOR signaling in aging tissue could be important for maintenance of muscle function.

## Future perspectives

Consistent with its role in coordinating protein synthesis, energy metabolism, and autophagy in cancer ^10, 139^, emerging evidence suggests that mTOR may act as a central node that orchestrates many aspects of cellular and organismal biology related to aging phenotypes ( Figure 2). Inhibition of the mTOR pathway by rapamycin or genetic means has profound effects on life span and age-associated phenotypes across a wide array of organisms. However, the underlying mechanisms are still unclear as it has been reported that during aging mTOR activity is both increased and decreased, depending on, for example, tissue or sex ^202, 243– 251^. It was suggested that, in spite of these variations, overall aging does not result in a generalized increase in mTOR signaling ^243^. If this is the case, it is possible that mTOR activity aligns with the antagonistic pleiotropy theory of aging ^252^, whereby its levels are beneficial during development but limit the health span in adult life.

Owing to its central role in age-related processes, mTOR represents an appealing target to ameliorate age-related pathologies. Despite its capacity to expand life span, the function of rapamycin (and of rapalogs) as an immunosuppressant ^3^ might be of concern, as a decline in immune function (immunosenescence) already occurs in the elderly, leading to infection-related morbidity and mortality ^253, 254^. Intriguingly, several studies in both mice and humans suggest that mTOR inhibitors could reduce immunosenescence ^202, 255^. In mice, rapamycin can restore the self-renewal and hematopoiesis of HSCs and enable effective vaccination against the influenza virus ^202^. A randomized trial testing the effects of rapalog RAD001 in a cohort of healthy elderly patients also showed an enhanced response to the influenza vaccination, accompanied by a reduction in programmed death 1 (PD-1) receptor expressing CD4 and CD8 T lymphocytes ^255^. PD-1 expression, which increases with age, inhibits T-cell signaling and reduces responses to antigen stimulation ^256^. Moreover, a pilot study concluded that short-term rapamycin treatment (8 weeks) in healthy older persons was safe ^257^.

Contrary to active-site mTOR inhibitors, allosteric inhibition of mTORC1 by rapamycin has little effect on 4E-BP phosphorylation ^22, 23^ and thus is expected to incompletely suppress mTORC1-dependent perturbations in translatome, mitochondrial functions, and metabolome ^10^. Indeed, studies in *D. melanogaster* showed that 4E-BP extends life span upon DR by enhancing mitochondrial activity ^258^. More recent studies showed that, in *D. melanogaster*, 4E-BP mediates temperature-induced effects on metabolism and life span ^259^ but that, in male mice, 4E-BP1 is involved in protecting against diet-induced obesity and insulin resistance ^260,261^. Both second generation of mTOR inhibitors that target its active site and third generation (combining allosteric and active-site inhibition; Rapalink-1) potently suppress 4E-BP phosphorylation ^7, 262^. Compared with what is known about rapamycin, however, much less is known of the effects of the active-site mTOR inhibitors in the context of aging.

Another limitation of rapamycin is that its chronic exposure in mice leads to mTORC2 inhibition in, for example, hepatocytes ^30, 31^. Active-site mTOR inhibitors also inhibit mTORC2 ^7^. Strikingly, selective suppression of mTORC2 reduces life span ^263, 264^ and is associated with changes in endocrinology and metabolism (for example, insulin resistance), which have a negative impact on health span ^31^. Thus, developing specific inhibitors which effectively suppress all mTORC1 outputs (including 4E-BP phosphorylation), but do not exert a major effect on mTORC2, appears to be warranted as a strategy to target age-related pathologies and improve health span. Interestingly, in a recent trial of healthy elderly patients, the combination of low-dose RAD001 (rapalog) and BEZ235 (dual mTOR/PI3K catalytic inhibitor) was proposed to selectively inhibit mTORC1 and not mTORC2 and led to enhanced immune function and a reduction in infections ^265^. However, it is important to note that complete inhibition of mTORC1 can be deleterious. Deficiency in RagA, a GTPase responsible for mTORC1 activation by nutrients, leads to loss of mTORC1 activity and is embryonic lethal in mice ^266^. Moreover, conditional deletion of RAPTOR (mTORC1 subunit) causes abnormalities in hematopoietic organs of adult mice ^267^. Given these serious side effects of total mTORC1 inhibition and in light of the positive data from the RAD001/BEZ235 low-dose trial, it would be tempting to speculate that incomplete mTORC1 inhibition achieved by intermittent or low-dose treatment with mTOR inhibitors (or both) would carry the benefits of mTORC1 inhibition while limiting the side effects ^268, 269^.

Biguanides (for example, metformin) are pharmaceuticals which are thought to have a beneficiary effect (in aging) that indirectly impinges on mTOR ^270^. Metformin is a first-line anti-diabetic drug which has been used for more than 60 years in the clinic and has very few side effects. It was shown to modulate life span in model organisms ( *C. elegans* or *M. musculus*), to affect several processes dysregulated in aging (for example, cellular senescence, inflammation, autophagy, and protein synthesis), and (with the exception of one study ^271^) to improve cognitive function and neurodegeneration in humans ^270^. At the organismal level, metformin reduces gluconeogenesis in the liver, which leads to normalization of glucose levels, decrease in insulinemia, and improvement of insulin resistance in fat, liver, and muscle ^272^. By inhibiting mitochondrial complex I, metformin causes energetic stress which results in mTORC1 inhibition through AMPK (5′ AMP-activated protein kinase)-dependent and independent mechanisms ^273^. Furthermore, metformin limits the secretion of numerous SASP factors by senescent cells ^274^ and has been shown to limit the spreading of cellular senescence *in vivo* ^191^. Although many studies have uncovered possible targets of metformin action in the cell in the context of aging, the full extent of metformin’s mechanism of action at the cellular and organismal levels is still incompletely understood. This is complicated by the issues of achievable drug doses in humans compared with the concentration used in cell culture and animal models ^272^. Nonetheless, clinical trials in which metformin is used to improve health span or aging-related conditions are being proposed. For instance, in the TAME (targeting aging with metformin) clinical trial, a placebo-controlled multi-center study of about 3000 elderly patients who are 65 to 79 years old, the effects of metformin on the development of age-associated outcomes like cardiovascular events, cancer, dementia, and mortality will be monitored ^270^. In addition to providing information about metformin efficacy, the TAME study could provide the basis of establishing aging as an indicator for therapeutic purposes, which may encourage the development of next-generation drugs that target aging and extend healthy life span by modulating mTOR.

Significant progress has been made toward understanding how the mTOR signaling pathway regulates cellular processes relevant to aging (cellular and organismal energetics, proteostasis, cell stemness, cellular senescence, and so on), but as many of these advances were made in the context of cancer, much less is known about how these regulations influence the fate of the aging cell. Notwithstanding that mTOR inhibition clearly extends life span, outstanding questions abound regarding the underpinning mechanisms. For example, is inhibition of mTORC1 (as opposed to mTORC2) the main driver of increased life span and health span? What is the extent to which mTOR inhibition mediates the positive effects of CR/DR? What is the role of the mTORC1-specific mRNA translation program in aging? Much work is still needed to fill many gaps in knowledge related to the function of mTOR in the context of aging. This work may uncover unappreciated regulators or pathways that control the aging process and could lead to the development of drugs aimed at improving the health of the aging population.

## Abbreviations

4E-BP, eukaryotic translation initiation factor 4E (eIF4E)-binding protein; AD, Alzheimer’s disease; AKT, protein kinase B; ATF4, activating transcription factor 4; ATF6, activating transcription factor 6; BCAA, branched-chain amino acid; CASTOR, cellular arginine sensor for mTORC1; COX-2, cyclooxygenase-2; CR, caloric restriction; DEPTOR, DEP domain-containing mTOR-interacting protein; DR, dietary restriction; eIF2, eukaryotic translation initiation factor 2; eIF2α , eukaryotic translation initiation factor 2 alpha; eIF2β, eukaryotic translation initiation factor 2 beta; eIF2B, eukaryotic translation initiation factor 2B; ERK, extracellular signal-regulated kinase; FLCN, folliculin; FNIP1, folliculin-interacting protein 1; FNIP2, folliculin interacting protein 2; GATOR1, GTPase activating proteins toward Rags 1; GATOR2, GTPase activating proteins toward Rags 2; GCN2, general control non-derepressible 2; GEF, guanine nucleotide exchange factor; HSC, hematopoietic stem cell; IGF, insulin-like growth factor; IIS, insulin/insulin-like growth factor 1 (IGF-I) signaling; IL, interleukin; IR, insulin receptor; IRE1, inositol-requiring enzyme 1; ISC, intestinal stem cell; KICSTOR, KPTN-, ITFG2-, C12orf66-, and SZT2-containing regulator of mTORC1; KO, knockout; LAMTOR, late endosomal/lysosomal adaptor, MAPK and MTOR activator; LRS, leucyl-tRNA synthetase; MAPK, mitogen-activated protein kinase; MBH, mediobasal hypothalamus; MDSPC, muscle-derived stem/progenitor cell; MTFP1, mitochondrial fission process 1; mTOR, mammalian/mechanistic target of rapamycin; mTORC1, mTOR complex 1; mTORC2, mTOR complex 2; NF-κB, nuclear factor kappa-light-chain-enhancer of activated B cells; NSC, neural stem cell; PD-1, programmed death 1; PERK, PKR-like ER kinase; PGC-1α, peroxisome proliferator-activated receptor gamma coactivator 1-alpha; PI3K, phosphatidylinositol-4,5-bisphosphate 3-kinase; PR, protein restriction; RAG, Ras-related GTP binding; RHEB, Ras homologue enriched in brain; S6K, ribosomal protein S6 kinase; SAMTOR, S-adenosylmethionine sensor for the mTORC1 pathway; SASP, senescence-associated secretory phenotype; SGK, serum and glucocorticoid-regulated kinase 1; SLC38A9, member 9 of the solute carrier family 38; TAME, targeting aging with metformin; TC, ternary complex; TSC, tuberous sclerosis complex; uORF, upstream open reading frame; UPR, unfolded protein response; v-ATPase, vacuolar-type H + ATPase; XBP1, X-box binding protein 1.
